# Carbonaceous Dye‐Sensitized Solar Cell Photoelectrodes

**DOI:** 10.1002/advs.201400025

**Published:** 2015-02-18

**Authors:** Munkhbayar Batmunkh, Mark J. Biggs, Joseph G. Shapter

**Affiliations:** ^1^ School of Chemical EngineeringThe University of AdelaideAdelaideSouth Australia5005Australia; ^2^ School of Chemical and Physical SciencesFlinders UniversityBedford ParkAdelaideSouth Australia5042Australia; ^3^ School of ScienceLoughborough UniversityLoughboroughLeicestershireLE11 3TUUK

**Keywords:** photovoltaic cells, dye‐sensitized solar cell, photoelectrode, carbon particle, carbon nanotube, graphene

## Abstract

High photovoltaic efficiency is one of the most important keys to the commercialization of dye sensitized solar cells (DSSCs) in the quickly growing renewable electricity generation market. The heart of the DSSC system is a wide bandgap semiconductor based photoelectrode film that helps to adsorb dye molecules and transport the injected electrons away into the electrical circuit. However, charge recombination, poor light harvesting efficiency and slow electron transport of the nanocrystalline oxide photoelectrode film are major issues in the DSSC's performance. Recently, semiconducting composites based on carbonaceous materials (carbon nanoparticles, carbon nanotubes (CNTs), and graphene) have been shown to be promising materials for the photoelectrode of DSSCs due to their fascinating properties and low cost. After a brief introduction to development of nanocrystalline oxide based films, this Review outlines advancements that have been achieved in the application of carbonaceous‐based materials in the photoelectrode of DSSCs and how these advancements have improved performance. In addition, several of the unsolved issues in this research area are discussed and some important future directions are also highlighted.

## 1 Introduction

The fact that only one‐thousandth of the Sun's energy incident on the Earth is equal to the entire world's current energy needs^1^ means direct conversion of this energy into electricity—photovoltaic (PV) energy—is now a mainstream renewable energy source.^2^ PV devices, or solar cells, have undergone considerable development over the past two decades: i) first generation silicon (Si) solar cells;^3^ ii) second generation solar cells based on semiconductor thin films;^4^ and iii) most recently, third generation solar cells represented by dye sensitized solar cells (DSSCs) and organic semiconductor solar cells.^5^, ^6^ While the first two generations are well established, their manufacture is inherently complex and expensive.^5^ The third generation cells such as DSSCs, on the other hand, are in principle far easier and cheaper to manufacture while also offering, at least in theory, greater efficiencies,^7^, ^8^, ^9^ although these have yet to be realized. Indeed, the highest standard configuration DSSC efficiency achieved to date is around 13%.^10^

**Figure 1:**
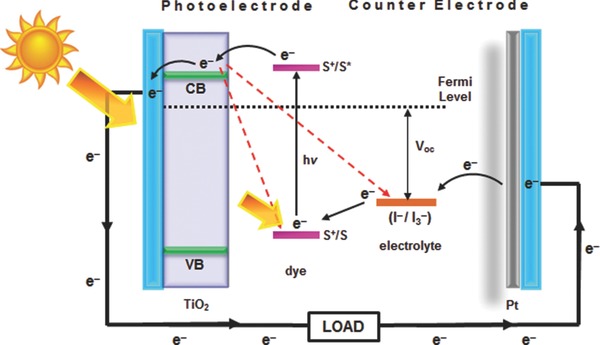
A schematic representation and principle of a typical DSSC with nanocrystalline TiO_2_ photoelectrode.

A typical DSSC consists of a metal‐oxide semiconductor electrode on which a photoactive dye is adsorbed (the photoelectrode), an electrolyte, and a counter‐electrode, as shown in **Figure** **1**.^11^, ^12^, ^13^ Upon exposure to photons, electrons from the dye molecules are excited and injected into the metal‐oxide electrode (i.e., the dye molecules are oxidized). These electrons then slowly diffuse through the metal‐oxide electrode before being conducted away through a power circuit to the counter‐electrode. The electrons then pass from the counter‐electrode into the electrolyte (i.e., the ions of the electrolyte are reduced), which in turn diffuses to the photoelectrode where it gives up the electrons to the dye molecules that have previously lost an electron to the circuit (i.e., they are regenerated). Of particular concern in this report is the photoelectrode.

In order to gain sufficient power, the photoelectrode of a DSSC is typically mesoporous so as to balance the need to maximize the density of adsorbed dye molecules while minimizing the resistance to electrolyte diffusion to the dye molecules. The most common (and original) mesoporous photoelectrodes are composed of Titania (TiO_2_) nanoparticles of around 20 nm in diameter deposited on a conductive transparent medium such as fluoride‐doped tin dioxide (FTO) glass. A variety of other nanostructured semi‐conducting films have, however, also been investigated, including those composed of zinc oxide (ZnO), tin oxide (SnO_2_) and niobium pentoxide (Nb_2_O_5_) nanoparticles.^14^, ^15^, ^16^ A significant issue with these nanostructured films is charge recombination arising from reaction between the photoexcited electrons that are slowly diffusing through them (towards the circuit) and the oxidized electrolyte species at that part of the electrode surface that happens to not be covered by dye molecules. This issue has led to some effort being focused on alternative photoelectrode materials, including those based on carbonaceous materials such as carbon particles, carbon nanotubes (CNTs) and, most recently, graphene. Therefore, review articles on carbon nanomaterials for the energy related applications are well documented.^17^, ^18^, ^19^, ^20^, ^21^, ^22^, ^23^, ^24^, ^25^, ^26^, ^27^, ^28^ It should be noted that since the production of this article, two other reviews of the use of graphene for DSSCs have been published.^29^, ^30^ The most recent one is very comprehensive and spans all aspects of DSSCs,^29^ while the other one briefly discussed the recent progresses of graphene based nanostructures in DSSCs.^30^ Here, we pay particular attention to the use of the complete spectrum of carbon materials and briefly cover some of the graphene work in the photoelectrodes of DSSCs. Following a brief overview of nanostructured DSSC photoelectrodes, we focus on the latest advancements that have been made on the utilization of carbonaceous materials in this context.

## 2 Development of Photoelectrodes in DSSCs

### 2.1 Nanostructured Photoelectrodes

**Figure 2:**
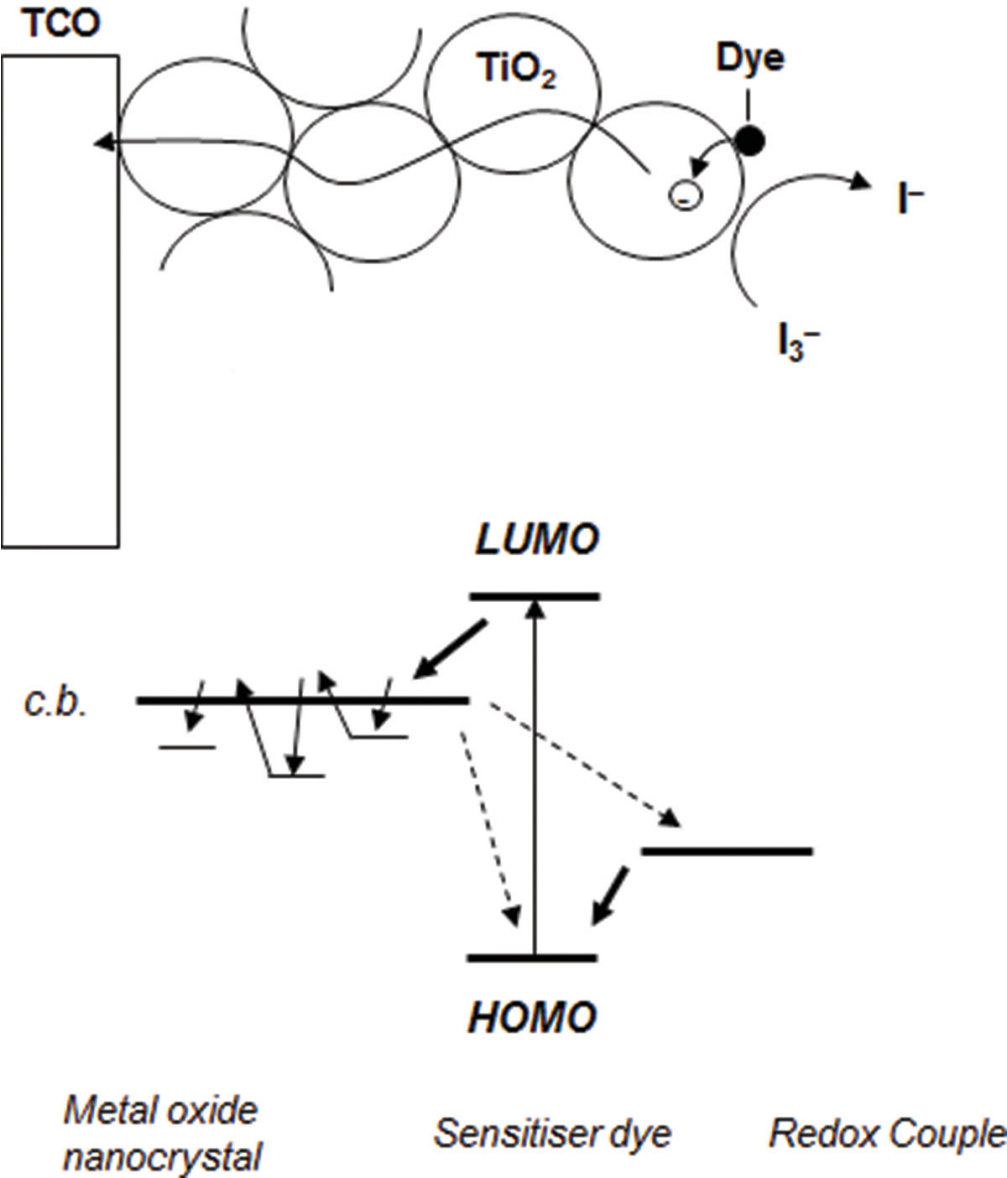
A schematic of electron transport in nanocrystallites based film. Electron trapping and detrapping process.^50^, ^51^ Reproduced with permission.^50^ Copyright 2004, Elsevier. Reproduced with permission.^51^ Copyright 2000, American Chemical Society.

In the early 1960s, metal oxide semiconductors with wide bandgap structures such as ZnO, TiO_2_, and SnO_2_ were used as photosensitizer materials.^31^, ^32^, ^33^ However, one major drawback of these wide bandgaps materials is their poor response to much of the solar spectrum. In particular, they only efficiently harvest the ultraviolet (UV) light, which constitutes around 2–3% of sunlight.^34^ This issue was eventually addressed by ‘sensitizing’ the semiconductors with dye molecules whose light absorption capacity lies in the visible region (i.e., wavelengths greater than 400 nm).^35^ By adsorbing dye molecules onto the oxides in this way, electrons excited in the dye by the sunlight can be injected into the conduction band of oxides (**Figure** **2**). The problem then was to adsorb a sufficient density of dye molecules to obtain the desired power – this was duly achieved by adopting thin (ca. 10 μm) mesoporous films of metal oxide nanoparticles,^36^ which possess relatively high surface area to volume ratios.

**Figure 3:**
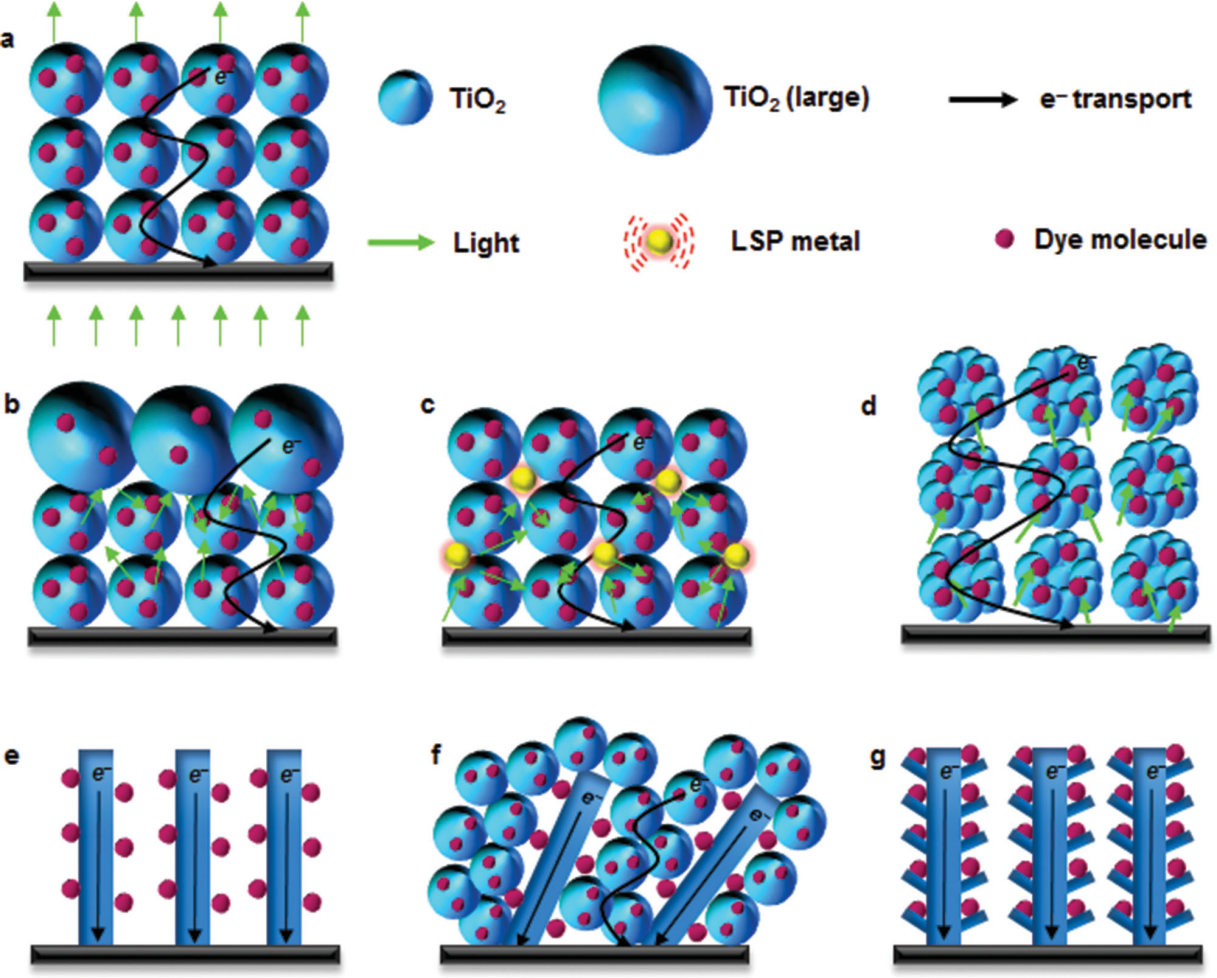
The structures of different photoelectrodes for DSSCs. a) Nanocrystalline TiO_2_ based photoelectrode film, b) double layer structured photoelectrode film, c) plasmon‐enhanced photoelectrode film, d) hierarchically structured nanoporous film, e) 1D structured photoelectrode film, f) 1D/nanoparticles hybrid structure based film, and g) hierarchically structured 1D photoelectrode.

Since the initial work of O'Regan and Graetzel, a range of n‐type metal oxide semiconductors have been investigated as alternatives to the TiO_2_ they used, including ZnO, SnO_2_, Nb_2_O_5_ and SrTiO_3_, all of which exhibit higher electron mobility than TiO_2_ while still being low cost and non‐toxic.^37^, ^38^, ^39^, ^40^ None have, however, replaced the nanostructured TiO_2_ (**Figure** **3**a) because surface area to volume ratios of these materials are lower than that of TiO_2_.^13^, ^41^, ^42^, ^43^ There are three primary factors that limit the performance of DSSCs fabricated based on these nanostructured semiconducting oxide materials: i) they are poor light harvesters because their constituent nanoparticles, which are smaller than the wavelength of the light, do not scatter the light;^44^, ^45^, ^46^, ^47^ ii) recombination is of major concern in the case of films consisting of nanocrystallites due to the fact that their size is several tens of nanometers and they are soaked in a liquid electrolyte with high ion concentration meaning they cannot support the required charge separation or facilitate a rapid electron transfer within the nanocrystallite network;^48^, ^49^ and iii) numerous grain boundaries between the nanoparticles and the diffusion of photo‐generated electrons in the nanocrystalline films suffer from random walk of electrons caused by a series of trapping and detrapping processes (Figure 2).^50^, ^51^ The electron trapping in the nanocrystalline film is a mechanism that causes significant energy loss. To date, several interesting approaches have been demonstrated to address these issues.^41^, ^44^, ^48^, ^52^ Here, the most important of these are briefly discussed.

One means of bringing about light scattering within the photoelectrode and, hence, improved interaction with the adsorbed dye is through the use of a bilayer structure as illustrated in Figure 3b.^53^, ^54^ Typically, the double layer structure consists of a layer of particles larger than the light wavelength (ca. 400 nm in size) being layered over the traditional film of small particles (ca. 20 nm in size).^55^ This layer of larger particles backscatters the light that passes through the layer of smaller particles so as it has a further opportunity to interact with the dye molecules adsorbed within it. This bilayer approach is an effective way to enhance the optical absorption of the photoelectrode, especially at wavelengths over 700 nm where the dye is not as efficient at absorbing light. Moreover, it is known that more than 40% of the total irradiance is absorbed in this wavelength region.^44^ Although the use of bilayer structure improves light collection efficiency, the large particles also bring a decrease in surface area and, hence, power generation capacity.

In the past few years, localized surface plasmon resonance (LSPR) of metal nanostructures has been considered a promising way to improve DSSC performance.^56^ Plasmonic noble metal nanostructures interact with light in the visible to near‐IR range through the creation of resonant surface plasmons. Several authors have seen significant improvement in the DSSC photocurrent by incorporating metal (Au, Ag) particles into semiconducting oxide nanoparticles (see Figure 3c).^57^, ^58^, ^59^, ^60^, ^61^ For example, Hou et al.^57^ observed a very high (2.4‐fold) enhancement in the PV efficiency compared to the conventional TiO_2_ film based DSSC due to the extension of light absorption over the wavelength range from 460–730 nm. However, the preparation method of homogeneous plasmonic nanocomposites involves a number of complex steps and high temperature & pressure, and the metal NPs are susceptible to corrosion by the electrolyte.^62^, ^63^

Hierarchical spherical nanostructures (HSN) such as that illustrated in Figure 3d have also been recently proposed as a means of simultaneously addressing the poor light harvesting efficiency of conventional DSSC films while boosting the surface area.^44^, ^64^ By using micrometer‐sized aggregates of nanosized particles, HSNs enhance the scattering of light within the films while retaining the area associated with the nanoparticles.^39^, ^44^, ^65^ The first study of such a bifunctional (high surface area to volume ratio and good light scattering property) structure in DSSCs was reported by Koo et al.^65^ who observed the amount of adsorbed dye was about 5 times greater than for film composed of similarly micro‐sized TiO_2_ particles. This leads to an energy conversion efficiency of 10.34%.^65^ Even though the HSNs remarkably improve both the light harvesting efficiency and adsorption of dye molecules into the film, the electrodes still suffer from charge recombination and slow electron transfer because they are composed of several small (20 nm) nanoparticles that cause electron trapping and detrapping.

The high rate of charge recombination and slow electron transfer in the nanocrystalline films increase the energy loss in the DSSC. In an effort to eliminate this issue, one dimensional (1D) nanostructures (see Figure 3e) such as nanotubes,^66^, ^67^ nanowires,^68^ nanorods,^69^, ^70^ and nanofibers^71^ have been proposed. The use of single crystal anatase TiO_2_ nanowires resulted in a photo conversion efficiency (PCE) of ca. 9.3%.^68^ While 1D materials lead to much more rapid transport of the electrons to the circuit, they suffer from low surface area to volume ratio due to their relatively large diameter^66^ (ca. 100 nm) and/or free space between them.^37^, ^52^

In order to address the low surface area to volume ratio and free space of 1D nanomaterials photoelectrode, a composite of 1D nanomaterials and nanoparticles such as that illustrated in Figure 3f have been proposed.^72^, ^73^ These composites not only ensure rapid electron transport and efficient use of space, they also enhance light scattering.^37^, ^48^ However, the PV performance (3.1%) obtained by this strategy was not as high as expected.^74^ This lower performance was, once again, attributed to the large number of grain boundaries between the 1D nanostructures and the spherical nanoparticles, leading to high electron recombination.^52^

In an effort to gain the advantages of 1D nanomaterials while avoiding the issues of poor volume utilization and excessive grain boundaries, Qu et al.^75^ have developed the hierarchical structure shown in Figure 3g.^75^ This structure fabricated using 1D hierarchical TiO_2_ yielded a PCE of 4.46%, far higher than that obtained from the 1D‐only structure in Figure 3f. Although 1D hierarchical TiO_2_ may fulfil many of the requirements of the ideal photoelectrode, the performance of the corresponding DSSC is still not high enough. Moreover, the synthesis of such structured TiO_2_ materials for the photoelectrodes uses complicated processes but still does not yield high performance. Very recently, due to their excellent conductivity, high electron mobility, low cost, good stability and abundance, carbonaceous materials have been considered good candidates for the photoelectrode of DSSCs. The detailed discussion of DSSCs fabricated with semiconducting composites based on the carbonaceous materials is presented in the following.

### 2.2 Carbonaceous Photoelectrodes

#### 2.2.1 Carbon Particles

A wide range of carbon nanomaterials have been utilized in DSSC application.^76^, ^77^, ^78^, ^79^, ^80^, ^81^, ^82^, ^83^ Among them, carbon black is one of the most commonly used materials for the counter electrode of DSSCs owing to its good electrical conductivity, catalytic activity, low cost and availability.^76^, ^81^ Although carbon blacks have been widely used in counter electrodes, they have been rarely used in DSSC photoelectrode.

**Figure 4:**
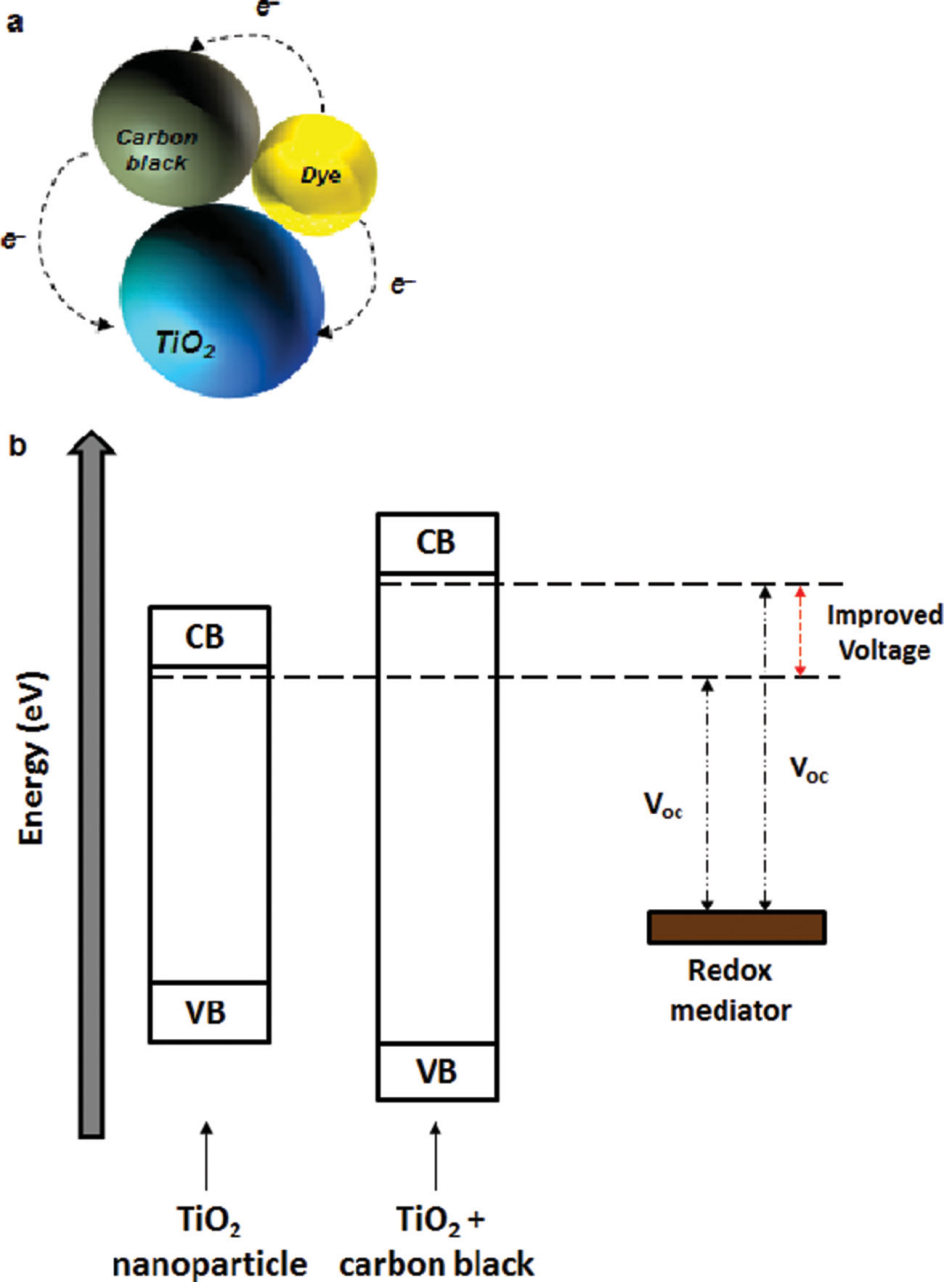
a) Schema of the TiO_2_ particle, carbon black and dye (triangular structure) and b) a possible mechanism for the V_oc_ improvement of TiO_2_ by adding carbon black. Figures are drawn based on the discussion of ref. 84.

Ting and Chao were the first to use carbon black in photoelectrodes.^84^ They used 22 nm diameter carbon particles as a bridge between nanocrystalline TiO_2_ and dye molecules in the photoelectrode of DSSCs (**Figure** **4**a). The open‐circuit voltage (V_oc_) of the cell improved after incorporating carbon black into TiO_2_ films. The authors hypothesized that this improvement is due to the increased energy level of TiO_2_ conduction band by adding carbon black (Figure 4b). It is well known that the V_oc_ in PV cells is mainly determined by the energy difference between the conductive band of semiconducting material and the potential energy of redox couple in the electrolyte (see Figure 1).^13^ However, the DSSC efficiency declined sharply when a high concentration of the carbon black is used. The authors suggested that this decrease in performance was due to the high loading of carbon particles in the photoelectrode films which interrupted the contact among TiO_2_, dye and electrolyte. In addition to this explanation, too much carbon black could decrease the light absorption of the window electrode and thereby limit the photoexcitation process. Indeed, the PCE (max. 0.17%) obtained in this study was relatively low as compared to the typical DSSCs because of the major replacement of each component.^84^

In order to effectively utilize the carbon particles in the photoelectrode of DSSCs, several researchers have used thermal treatment processes on the carbon powder incorporated TiO_2_ film.^85^, ^86^, ^87^ By using this method, these authors prepared highly porous structured films with improved surface area for high dye loading and light scattering ability. In Kang et al,^85^ after applying thermal treatment on carbon/TiO_2_ electrode, a considerable improvement (max. ca. 31%) in the surface area of the film was observed as compared to a TiO_2_ only film. Because of this improved surface area, they achieved a high energy conversion efficiency of 5.65% using DSSCs fabricated with 1 wt% (optimized content) carbon particles added to the TiO_2_ film. This optimum concentration of the carbon powder in TiO_2_ film was further confirmed by Kim et al.^86^ who also prepared nanoporous carbon/TiO_2_ films using a hydrothermal method for use as the photoelectrode in DSSCs. The efficiency of their carbon/TiO_2_ photoelectrode based DSSC was about 3.4% which was higher than that (2.5%) of the reference device.

Yang et al.^87^ synthesized spherical carbon particles with three different sizes (diameters of 250 nm, 500 nm, and 700 nm) using a hydrothermal method and incorporated them into nanocrystalline TiO_2_ films. After sintering the films at high temperature, the carbon spheres were burned out and thus holes were formed corresponding to the size of initial carbon spheres. The authors studied the influence of hole sizes made in the films on the light absorption characteristics for DSSC performance. The sequence of the light scattering ability of these films was C500 > C700 > C250 > C0. Due to the higher light scattering ability of the C500 film, a 26.5% improvement in the J_sc_ (when compared to a TiO_2_ nanocrystalline only film based device) was achieved using photoelectrodes based on the 500 nm carbon spheres. A poor J_sc_ obtained by DSSCs with the large holes (700 nm) was due to the decreased amount of dye in the film. Indeed, by balancing the light absorption and dye adsorption ability of the film, the highest efficiency was 7.2% achieved by the cell fabricated with 500 nm carbon particles, while the standard cell reaches 5.6% efficiency.

**Figure 5:**
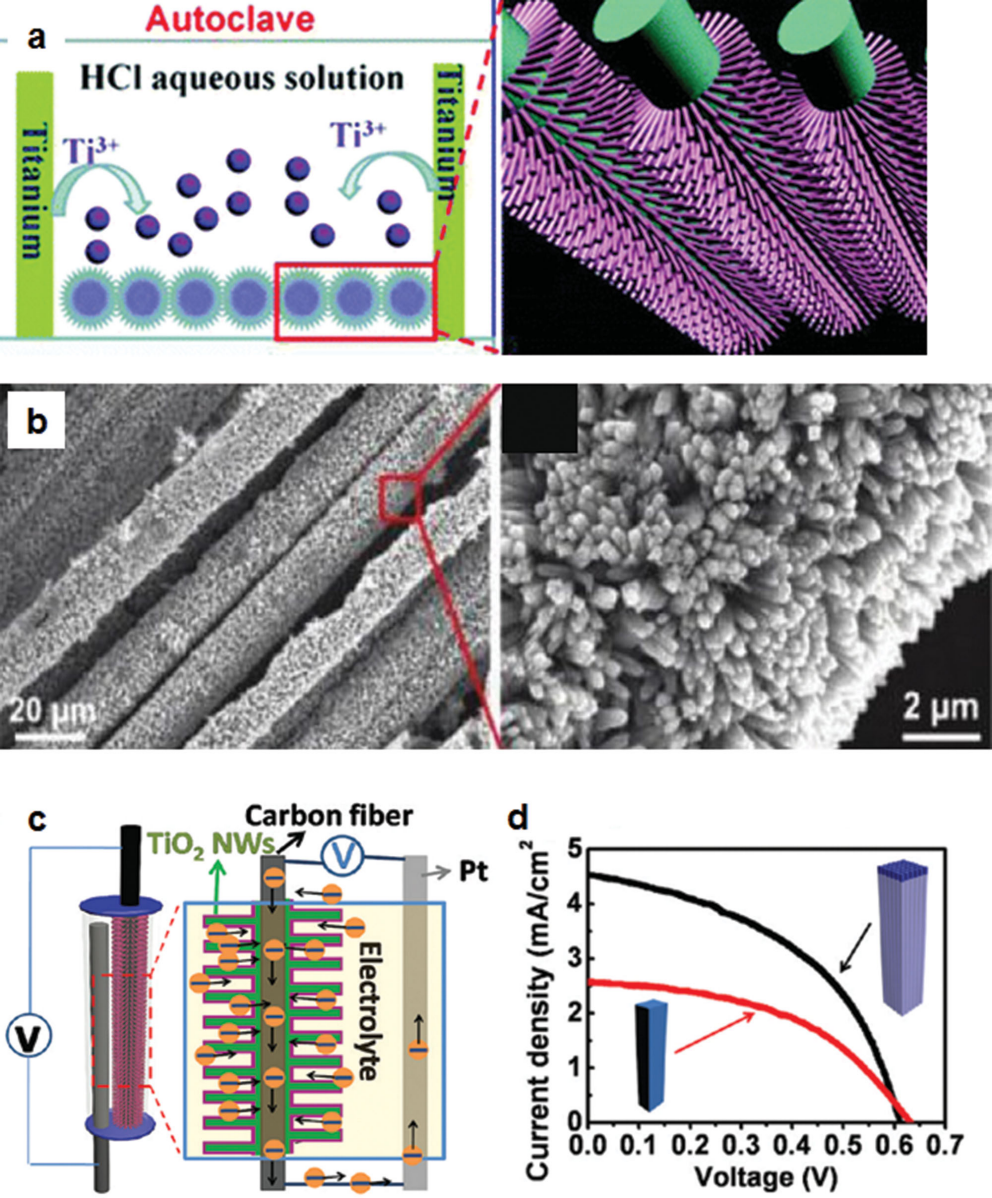
a) A schematic illustration of the growth of rectangular bunched TiO_2_ NRs on CFs, b) SEM of 3D structure formed with TiO_2_ NRs@CFs, c) DSSC fabricated with 3D structured photoelectrode, and d) current density–voltage (J–V) curves of DSSCs fabricated with the TiO_2_ NRs and TiO_2_ NRs@CFs.^89^ Reproduced with permission.^89^ Copyright 2012, American Chemical Society.

Carbon fibres (CFs) are cylindrical structures with graphene layers arranged as stacked cones, cups, ribbons or plates. In the past few years, CFs have been used in the photoelectrode of DSSCs due to their good conductivity, low weight and high stability.^88^, ^89^ Moreover, the cylindrical shape of CFs is also expected to promote the electron transport within the film.^90^ Recently, Guo et al.^89^ synthesized rectangular bunched TiO_2_ nanorod (NR) arrays using a hydrothermal approach. This structure was vertically aligned on the CFs to build the photoelectrode of DSSCs. The preparation route of NRs on the CFs is shown in **Figure** **5**a. This synthesis method of the CFs with TiO_2_ NRs is called a “dissolve and grow” process. In the resulting structure (Figure 5b), the rectangular bunched TiO_2_ NRs (as termed by the authors) were designed to simultaneously address the poor dye loading of a 1D structure and the light capturing ability of TiO_2_ nanocrystalline film. Therefore, the bunched NRs/CFs structured photoelectrode exhibited an improved surface area, which enabled more dye molecules to be adsorbed. With the 3D structured photoelectrode made using the carbon fibres (Figure 5c), the conversion efficiency of DSSC reached 1.28%, which was ca. 68% higher than that of the NRs‐only (see Figure 5d).

Application of carbon structures in TiO_2_ nanocrystalline based films is a good strategy that can suppress the charge recombination using a highly conductive carbon layer. A graphitic thin film embedded (referred as carbonized) with semiconducting oxide particles was prepared for use as the photoelectrode of a DSSC.^91^ In Jang et al,^91^ three different (carbon layer under, on or both under and on the film) carbonized nanocrystalline TiO_2_ films were fabricated. By introducing graphitic carbons into the TiO_2_, the amount of dye loading was decreased slightly due to the reduced surface area of the film. Although the adsorption of dye molecules was reduced, the embedded carbons in the TiO_2_ film improved the electron recombination lifetimes (τ_r_) of DSSCs significantly because of their high conductivity. Due to this improved property of the cells, carbonized TiO_2_ films based DSSCs achieved very high current densities (**Table** **1**). It was noted by these authors that the surface area of the films in such structured device plays a minor role for the PV performance.^91^ Finally, a 40.6% improvement (as compared to the reference cell) in the PV efficiency was obtained by DSSC fabricated with both parts (under and on top) carbonized TiO_2_ thin layers.

**Table 1:** PV and electrochemical characteristics of four different DSSCs fabricated in the literature

Photoelectrode	Dye amount, (mmol*cm^−2^)	R_ct_ (Ω)	τr (ms)	Jsc (mA*cm^−2^)	PCE (%)
TiO_2_–only film	7.19 × 10^−5^	74.47	5.1	6.58	3.21
Lower part carbonized film	6.09 × 10^−5^	64.02	22.1	6.94	3.71
Upper part carbonized film	5.78 × 10^−5^	52.10	25.3	8.96	4.91
Both parts carbonized film	5.47 × 10^−5^	51.84	29.6	9.35	5.21

Data points are collected from ref. 91.

**Figure 6:**
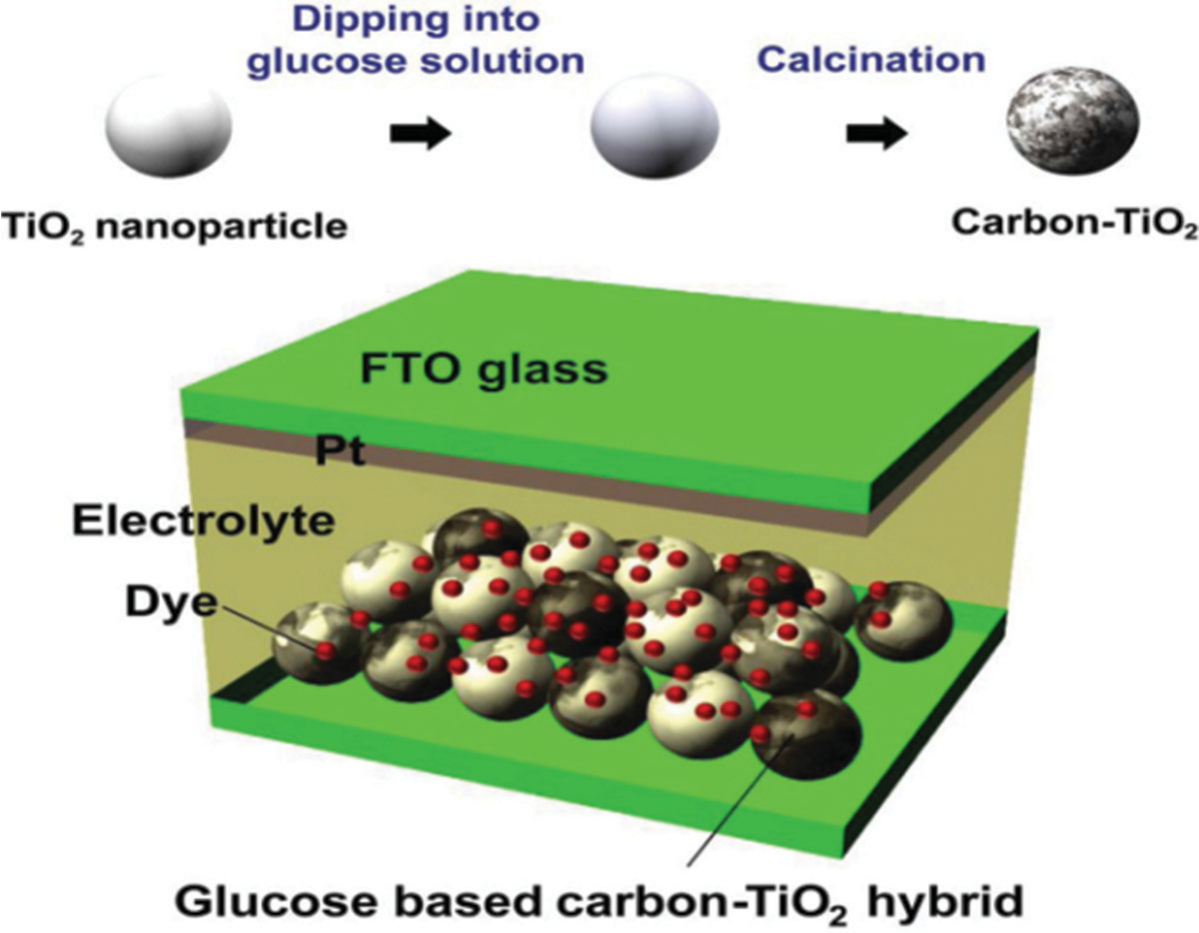
Configuration of DSSC fabricated with glucose–based carbon/TiO_2_ film.^94^ Reproduced with permission.^94^

Carbon particles can be prepared from sucrose, glucose and starch which are generated by the polymerization and aromatization of carbohydrate molecules. The carbohydrates are mostly converted into carbons using a hydrothermal method under certain conditions.^92^, ^93^ Preparing carbons from the carbohydrates has many advantages including a lack of toxicity, a facile synthesis process, use of relatively low temperature coupled with economic viability. Due to these advantages, Jang and co‐workers used a glucose‐based carbon incorporated TiO_2_ photoelectrode film (see **Figure** **6**) for DSSC.^94^ The J_sc_ and PCE of the DSSCs containing glucose/TiO_2_ photoelectrode were increased by 20.9% and 11.6%, respectively, as compared to those of the conventional DSSC. The improved performance by adding glucose‐based carbon was proven to be due to the improved charge transport within the photoelectrode. However, the cell efficiency was significantly decreased when a high concentration of carbons were used because the presence of large amount of carbons acted as a competitor of dye molecules in light harvesting.

#### 2.2.2 Carbon Nanotubes (CNTs)

As shown by red dash arrows in Figure 1, charge recombination and/or back electron transfer are the most pressing problems for the improvement in DSSC efficiency. There are mainly two possible recombination routes in DSSCs: the direct recombination of electrons from the conduction band of semiconducting TiO_2_ to the oxidized dyes or to the electrolyte. The frequency of the electron recombination to the dye molecule is in the order of a micro to millisecond, whereas that to the electrolyte is in the range of a millisecond to second. Both these recombinations take place at the TiO_2_/dye and TiO_2_/electrolyte interface. It has been established that these recombinations can be suppressed by using 1D nanostructures based photoelectrodes. In this regard, as a first member of 1D structures, CNTs are very promising candidates for the DSSC photoelectrodes due to several of their extraordinary properties. Notably, CNTs not only benefit from the 1D structure that provides fast electron transport pathway, their highly conductive character also plays a critical role in DSSCs.

**Figure 7:**
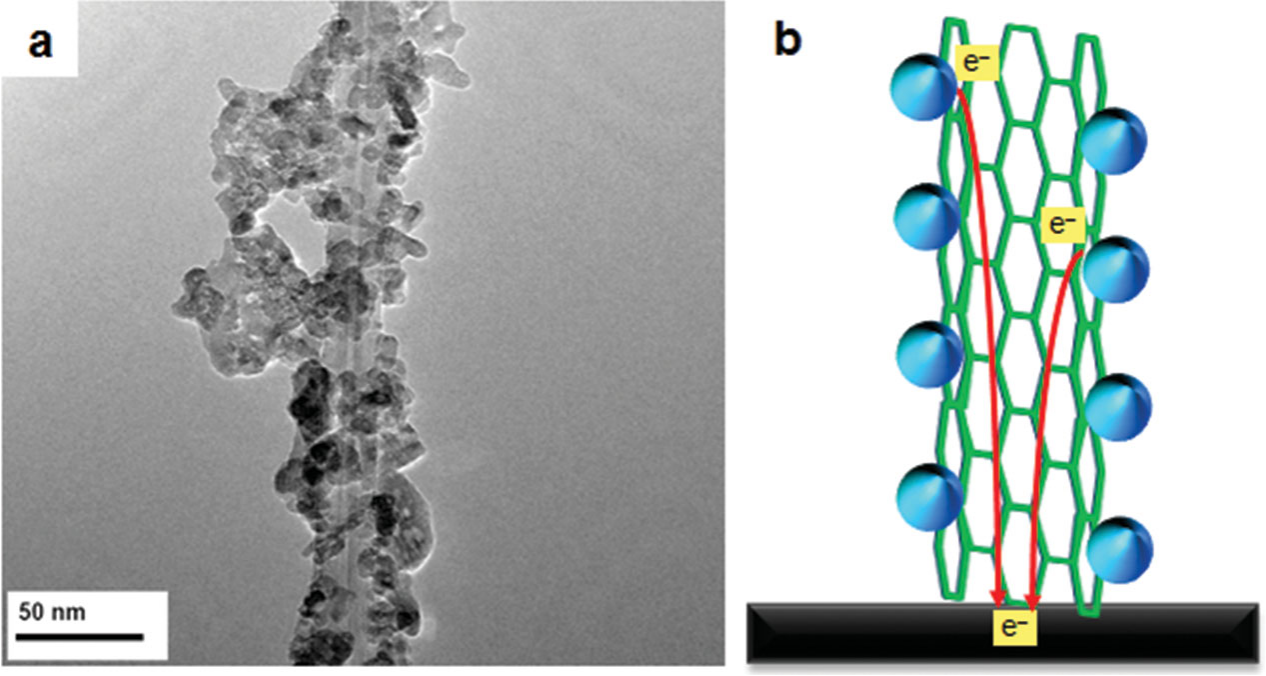
a) TEM image of MWCNTs‐TiO_2_ composite,^99^ and b) schematic diagram for electron transfer in the CNTs‐TiO_2_ film. Part (a) reproduced with permission.^99^ Copyright 2011, Elsevier.

Because of their high charge mobility and/or excellent electrical conductivity that can decrease the charge transfer resistance (R_ct_) of films, CNTs were expected to improve the performance of DSSCs. In 2004, Jang et al.^95^ were the first to report using CNTs in the photoelectrode of DSSCs and they achieved a 25% increase in the J_sc_ compared to the CNTs–free cell. Since this significant improvement in the DSSC performance was demonstrated by these authors using CNTs, considerable attention has been paid to the research on this topic.^96^, ^97^, ^98^, ^99^, ^100^, ^101^, ^102^, ^103^, ^104^, ^105^, ^106^, ^107^, ^108^, ^109^ For instance, Lin et al.^99^ prepared bilayer structured photoelectrode films composed of multi‐walled carbon nanotubes (MWCNTs)–TiO_2_/TiO_2_ which when used in DSSCs exhibited two times higher PV efficiency than the cell fabricated with the bare TiO_2_ film. This improvement in the PV performance has been demonstrated to be related to the 1D CNT which supports transfer of the photo‐generated charges quickly, thus suppressing charge recombination. **Figure** **7**a depicts the complete attachment of TiO_2_ to the CNT surface. The injected electrons from the excited dye molecules into the conduction band of TiO_2_ can be transferred quickly through the CNTs conduit, as expressed in Figure 7b. Furthermore, Chen et al.^100^ confirmed that the electrical conductivity of the bare TiO_2_ films can be significantly improved by incorporating the CNTs structure into TiO_2_ nanocrystalline films. Although the conductivity of the films can be improved by incorporating higher CNTs content, the opaqueness and light absorbing properties of CNTs with high concentration ultimately decreases the incident photon‐to‐conversion efficiency (IPCE) of the film electrodes. Therefore, it is very important to pursue the right content of CNTs structures in TiO_2_ films that can optimize the conductivity and light harvesting efficiency of the electrode.

In order to obtain a balance between the R_ct_ and IPCE, several efforts have been undertaken with different concentrations of CNTs structures.^97^, ^100^, ^101^, ^102^, ^103^, ^104^ In these studies, the optimized concentrations of CNTs in TiO_2_ films were relatively different because the corresponding DSSCs were fabricated under different experimental conditions. A general method to prepare CNTs/TiO_2_ photoelectrodes is as follows: CNTs are first chemically treated using acid solutions (HNO_3_ or H_2_SO_4_) to generate functional groups such as hydroxyl (–OH), carbonyl (C=O) and carboxyl (–COOH) groups. Then, the functionalized CNTs are mixed with nanocrystalline TiO_2_ nanoparticles, followed by a drying process under certain temperature to prepare CNTs/TiO_2_ pastes. Finally, the obtained paste can be either deposited on transparent conducting oxide substrates via doctor blade technique or screen printing technique. By applying this method, Yu et al.^101^ prepared CNTs/TiO_2_ based photoelectrodes with various concentrations of CNTs (0–1.0 wt%) and compared the efficiencies of the devices. As a result, a maximum conversion efficiency of 4.5% was obtained for a DSSC with a photoelectrode with 0.2 wt% CNTs incorporated into the TiO_2_ film. Furthermore, a similar observation has been made by Chen et al.^100^ who prepared MWCNTs/TiO_2_ composite films with CNTs concentrations of 0, 0.021, 0.043, 0.086, 0.172, 0.258, and 0.343 wt%. The DSSC made using a 0.172 wt% CNTs/TiO_2_ based photoelectrode gave the highest efficiency of ca. 5.2%. On the other hand, some studies showed that to obtain the best DSSC performance the concentration of CNTs in TiO_2_ film should be around 0.01–0.03 wt%.^102^, ^103^, ^104^ These different optimized contents in these case studies are mainly due to the fact that those CNTs were not functionalized using chemical acids prior to incorporating into TiO_2_ films. By comparing these results reported in the literatures,^97^, ^100^, ^101^, ^102^, ^103^, ^104^ it can be concluded that the optimal content of the functionalized CNTs in TiO_2_ films varies from 0.1 to 0.3 wt% depending on the acid–functionalization level.

**Figure 8:**
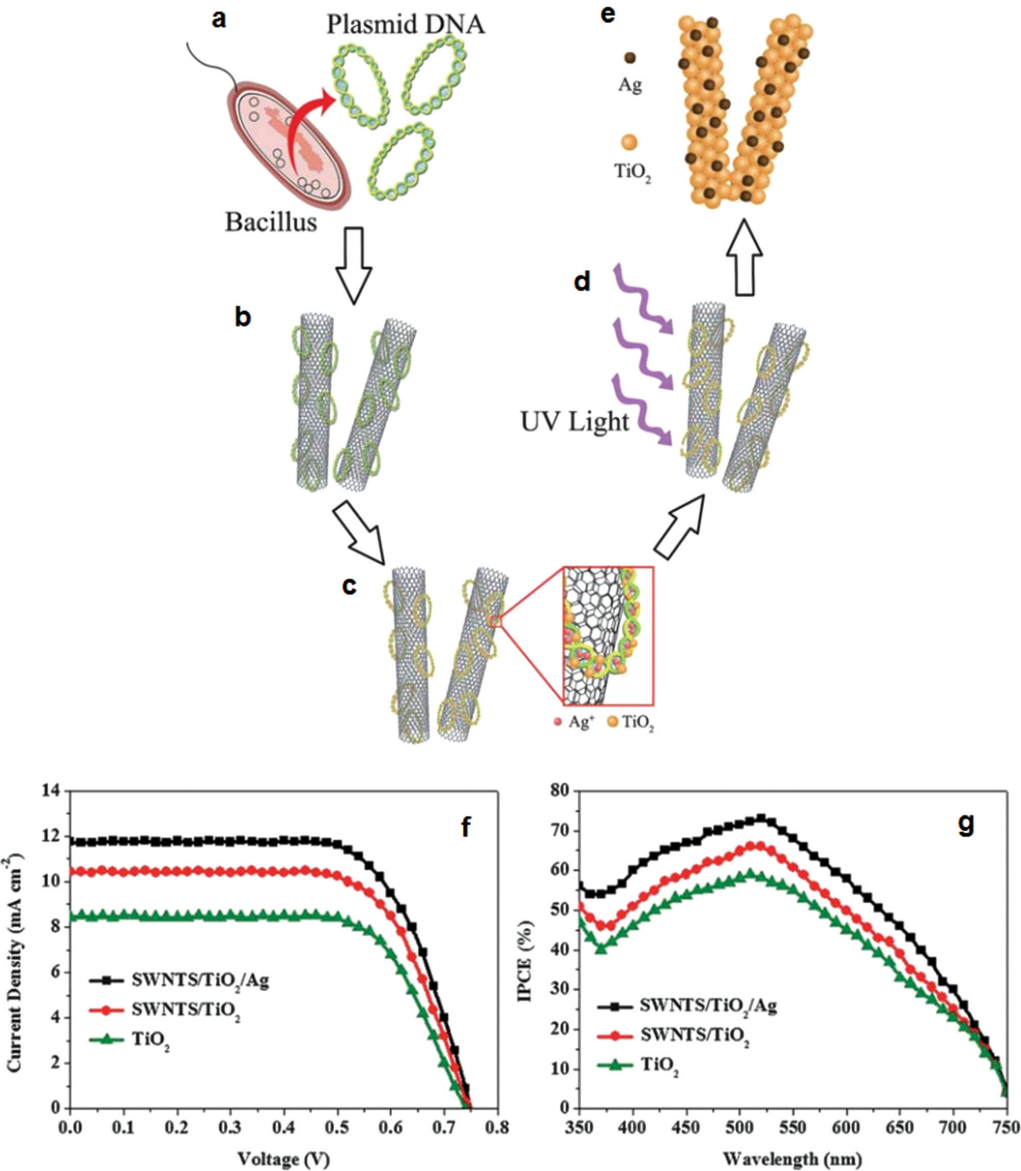
a–e) Schematic illustration of the synthesis process of s‐SWCNTs/TiO_2_/Ag nanocomposite for the DSSC photoelectrode, f) J–V curves and g) IPCE spectra of DSSCs fabricated with TiO_2_‐only, s‐SWCNTs/TiO_2_ and s‐SWCNTs/TiO_2_/Ag photoelectrodes.^106^ Reproduced with permission.^106^ Copyright 2013, Royal Society of Chemistry.

The performance of DSSCs containing CNTs materials strongly depends on the dispersion of CNTs in a base fluid.^105^, ^106^ It has been established that pristine CNTs are difficult to disperse in base fluids (distilled water, anhydrous ethanol, etc.), which could be due to a large aspect ratio and lack of hydrophilic groups.^110^ Therefore, enhanced spatial distribution and improved dispersibility of CNTs in the solvents are the key requirements to obtain the excellent properties of CNTs. Recently, Zhang et al.^106^ introduced DNA as a biological scaffold on semiconducting single‐walled carbon nanotubes (s‐SWCNTs) network in order to upgrade the dispersibility of CNT solution. The upgraded s‐SWCNTs dispersion was then utilized to integrate the s‐SWCNTs/TiO_2_ composite for the use in photoelectrode films. In addition, they also added plasmonic metallic silver nanoparticles (AgNPs) into the s–SWCNTs/TiO_2_ film to further improve the performance of DSSCs. The synthesis process of the s‐SWCNTs/TiO_2_/AgNPs nanocomposite is illustrated in **Figure** **8**a–e. In this work,^106^ the energy conversion efficiency of the DSSC increased from 4.37% to 5.32% after adding 0.15 wt% s‐SWCNTs compared to the TiO_2_‐only photoelectrode system. Furthermore, the DSSC fabricated with s‐SWCNTs/TiO_2_/AgNPs photoelectrode exhibited the highest efficiency of 5.99% due to the improved electron collection and transportation by s‐SWCNTs, and the enhanced light‐harvesting efficiency by plasmonic AgNPs (see Figure 8f and 8g).

**Figure 9:**
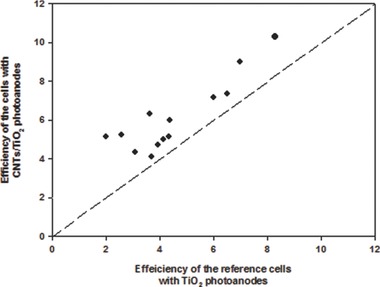
PV efficiencies of DSSCs fabricated with and without CNTs in the TiO_2_ films. Data obtained from refs. 97, 98, 99, 100, 101, 102, 103, 104, 105, 106, 107, 108, 109.

Several researchers have used CNT materials in TiO_2_ photoelectrode films to boost the PV efficiency of DSSCs.^96^, ^97^, ^98^, ^99^, ^100^, ^101^, ^102^, ^103^, ^104^, ^105^, ^106^, ^107^, ^108^, ^109^ It can be clearly seen from **Figure** **9** that the recorded efficiencies of CNTs/TiO_2_ photoelectrodes based DSSCs vary from 4.1% to 10.6% depending on the experimental conditions and applied techniques. So far, the best efficiency of CNTs/TiO_2_ photoelectrode based DSSC has been achieved by Dang et al.^107^ who introduced multiple genes of a virus into s‐SWCNTs based aqueous solution. The prepared pastes composed of virus/s‐SWCNTs/TiO_2_ were deposited onto FTO glass substrates using a doctor blade technique. As a result, the observed J_sc_, V_oc_ and FF for DSSC fabricated with 0.1 wt% s‐SWCNTs/TiO_2_ composite film were 20.3 mA cm^−2^, 0.78 V and 0.7, respectively, and yielded a very high energy conversion efficiency of 10.6%. Interestingly, these authors observed a 27% improvement in the J_sc_ when s‐SWCNTs were used; whereas the J_sc_ was decreased by ca. 20% after adding a pure metallic SWCNTs (m‐SWCNTs), as compared to only TiO_2_ based DSSC.

**Figure 10:**
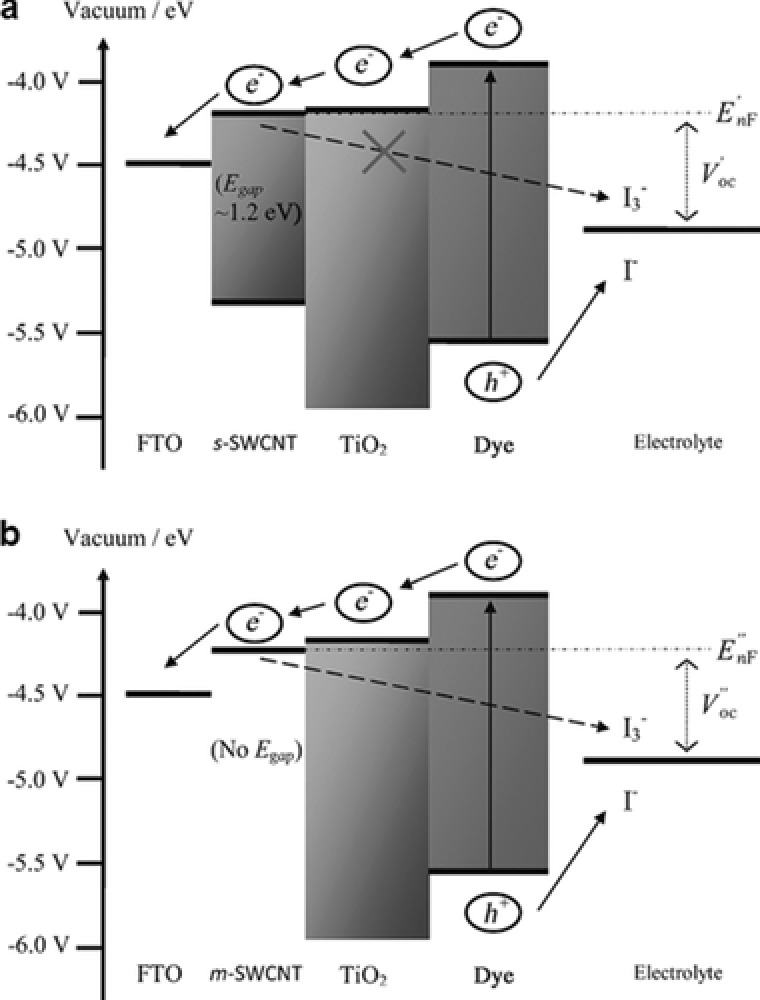
A schematic representation of the energy diagram of DSSCs with a) s‐SWCNTs and b) m‐SWCNTs added TiO_2_ films.^108^ Reproduced with permission.^108^

Guai et al.^108^ later showed a similar finding to that of Dang et al^107^ namely that the s‐SWCNTs suppress the charge recombination in DSSCs and thereby enhance the overall efficiency. The improved performance of DSSC was because of the increased electron diffusion length by s‐SWCNTs, leading to higher electron collections. Notably, the s‐SWCNTs possess a non‐continuous band structure, while the m‐SWCNTs have zero bandgap.^107^ So, in the 3D networks of s‐SWCNTs/TiO_2_, the electrons, transferred from the conduction band of TiO_2_, can be transported to the conducting oxide film without charge recombination because the higher energy barrier of s‐SWCNTs compared to m‐SWCNTs blocked the back flow of dye‐injected electrons to the electrolyte (see **Figure** **10**a). For the case of the m‐SWCNTs, although they can transport the photoelectrons more rapidly due to higher mobility than s‐SWCNTs, the charge transport was disrupted with an increased back electron transfer to the electrolyte (Figure 10b).

SWCNTs can be metallic or semiconducting with bandgaps ranging from 0 to 2.0 eV depending on their diameter, level of defects or functionalization, and degree of aggregation.^111^ Based on this concept, it should be possible to improve the efficiency of DSSCs by optimizing the bandgap energy of s‐SWCNTs. Therefore, systematically exploring the influence of different bandgap energies of s‐SWCNTs on the performance of DSSCs would be of great value.

#### 2.2.3 Graphene

Graphene^112^—a single layer of carbon atoms arranged in a hexagonal lattice—is a material that possesses remarkable properties including excellent conductivity, superior strength to any material ever isolated, good flexibility, high transparency and chemical resistivity.^113^, ^114^, ^115^, ^116^, ^117^ The 2010 Nobel Prize in physics was awarded to Andre Geim and Konstantin Novoselov for their discovery of the unique properties of graphene.^112^ Since then, graphene has become known the world‐over as an advanced material and is quickly moving from the research laboratories to the industrial applications.^118^ The exceptional properties of this material have pioneered recent explorations to apply graphene structures in the photoelectrode of DSSCs.^119^, ^120^, ^121^, ^122^, ^123^, ^124^, ^125^, ^126^, ^127^, ^128^, ^129^, ^130^, ^131^ It can be clearly seen from **Table** **2** that the improved efficiencies of DSSCs with graphene materials incorporated TiO_2_ photoelectrode films vary from 1.68% to 8.13%. These differences in the cell performances are possibly due to the utilization of different experimental conditions such as the active area of the cells, type of dyes, film preparation methods and various treatments (see Table 2).

**Table 2:** PV characteristics of different DSSCs fabricated under various conditions. Graphene, modified Hummers method^132^ and hydrazine are abbreviated as “G”, “MH method” and “hyd”, respectively. The abbreviation of “**↑↑**” and “**↓**” in the dye adsorption column represents the amount of adsorbed dye in the rGO/TiO_2_ film “increased” and “decreased”, respectively, as compared to TiO_2_‐only film

Photoelectrode film	J_sc_, mA*cm^−2^	PCE,%	Cell area, cm^2^	Dye type	Deposition method	Synthesis method of “G”	Treatment	“G” conc	Dye loading	Ref
rGO/TiO_2_	16.29	6.97	0.2	N3	doctor blade	MH method – hyd & thermal reduction	poly vinyl–alcohol	0.6 wt%	–	121
TiO_2_	11.26	5.01								
rGO/TiO_2_	6.67	1.68	0.5	N719	electro‐phoretic	MH method – hyd reduction	molecular grafting	–	**↑↑**	122
TiO_2_	1.95	0.32								
G/G/TiO_2_	19.47	8.13	0.25	N719	doctor blade	MH method – hyd and thermal reduction	‘G’+TiCl_4_ coated FTO	–	**↓**	123
TiO_2_	15.2	5.8								
rGO/TiO_2_	13.5	7.25	0.2	N719	doctor blade	MH method – solvothermal reduction	ultrathin TiO_2_ NRs	–	**↑↑**	124
P25 TiO_2_	6.2	2.85								
rGO/TiO_2_	14.8	6.49	0.15	Indoline	doctor blade	MH method – hyd and hydro‐thermal reduction	multilayer film	–	**↑↑**	125
P25 TiO_2_	11.9	4.96								
rGO/TiO_2_	13.93	7.1	–	N719	doctor blade	MH method –	in situ reduction‐hydrolysis	–	**↓**	126
TiO_2_	10.99	5.3				high thermal reduction				
rGO/TiO_2_	7.6	2.78	0.5 × 1.0	N719	doctor blade	MH method – thermal reduction	–	0.83 wt%	**↓**	127
TiO_2_	4.96	1.79								
rGO/TiO_2_	16.8	5.77	0.4	N719	doctor blade	–	pre‐treated TiO_2_	0.75 wt%	–	128
TiO_2_	13.7	4.61								
rGO/TiO_2_	12.16	5.5	0.5	N719	screen print	MH method – solvothermal reduction	GO in ethylene glycol	0.75 wt%	–	129
TiO_2_	10.75	4.2								
G/TiO_2_	19.92	6.86	–	N719	spin coating	–	Addition surfactant	1.0 wt%	**↑↑**	130
TiO_2_	18.83	5.98								
rGO/TiO_2_	18.2	6.06	0.16	D9	doctor blade and spray coat	MH method – hyd and thermal reduction	rGO was coated on TiO_2_ film	–	**↓**	131
TiO_2_	16.4	5.09								

**Figure 11:**
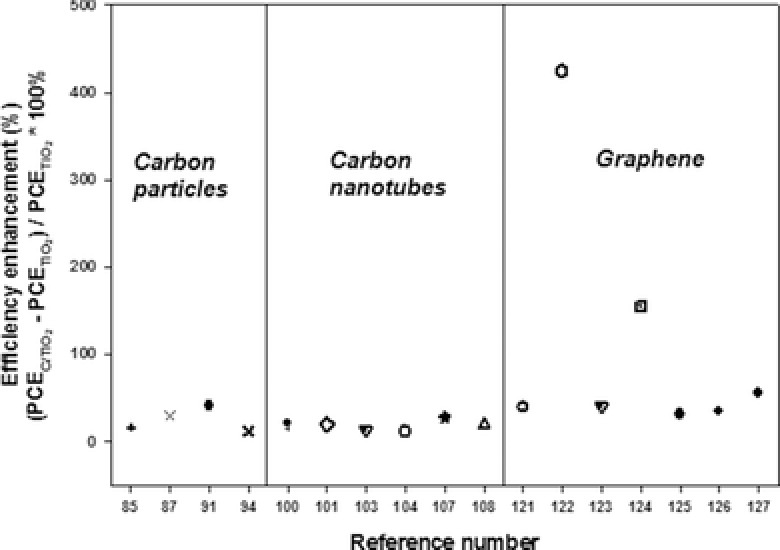
Efficiency enhancements of the DSSCs with carbon particles, CNTs and graphene incorporated TiO_2_ films. Data points are adopted from refs. 85, 87, 91, 94, 100, 101, 103, 104, 107, 108, 121, 122, 123, 124, 125, 126, 127.

Moreover, as listed in Table 2, the efficiencies recorded for the conventional DSSCs also vary considerably ranging from 0.32% to 5.8%, despite all the cells being made very similarly (TiO_2_ photoelectrode film, Ruthenium based organic dye, iodolyte electrolyte and Pt counter electrode). Because of these varying performances, it is difficult to compare the improvements that have been achieved by the use of graphene structures. To better understand the real enhancement of DSSCs performance obtained by applying carbonaceous materials based films, the efficiency enhancements are calculated and plotted in **Figure** **11**.

Figure 11 shows the efficiency enhancements (%) of various DSSCs fabricated using carbon particles, CNTs and graphene incorporated TiO_2_ photoelectrode films. One can simply observe from Figure 11 that the average enhanced efficiencies obtained by graphene/TiO_2_ photoelectrode based DSSCs is higher than those achieved by DSSCs with carbon particles and CNTs based TiO_2_ films. There are several reasons that can be given to explain this observation that graphene improves the performance of cells more compared to other carbonaceous materials.

**Figure 12:**
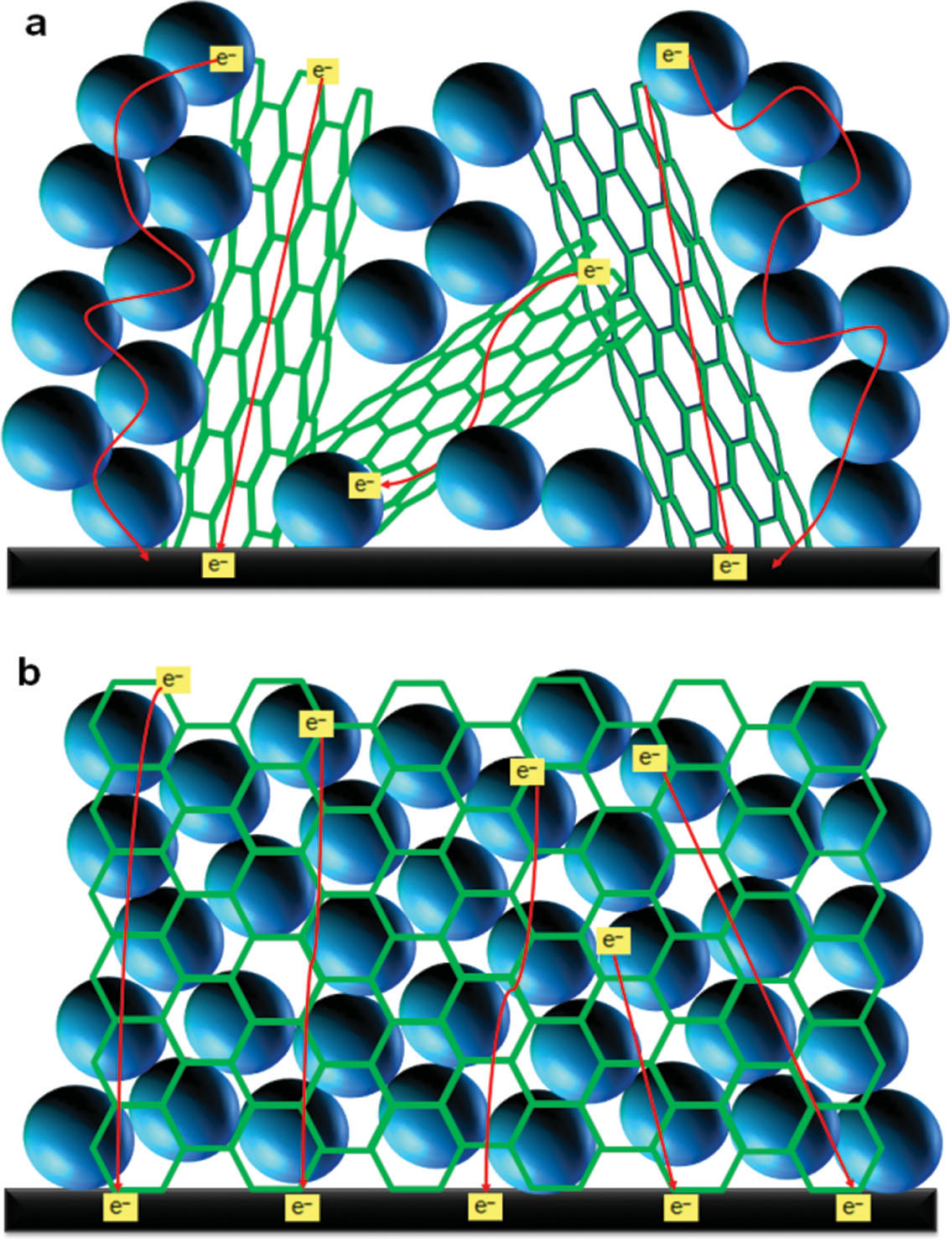
Schematic representation of a) CNTs/TiO_2_ and b) graphene/TiO_2_ films.

**Figure 13:**
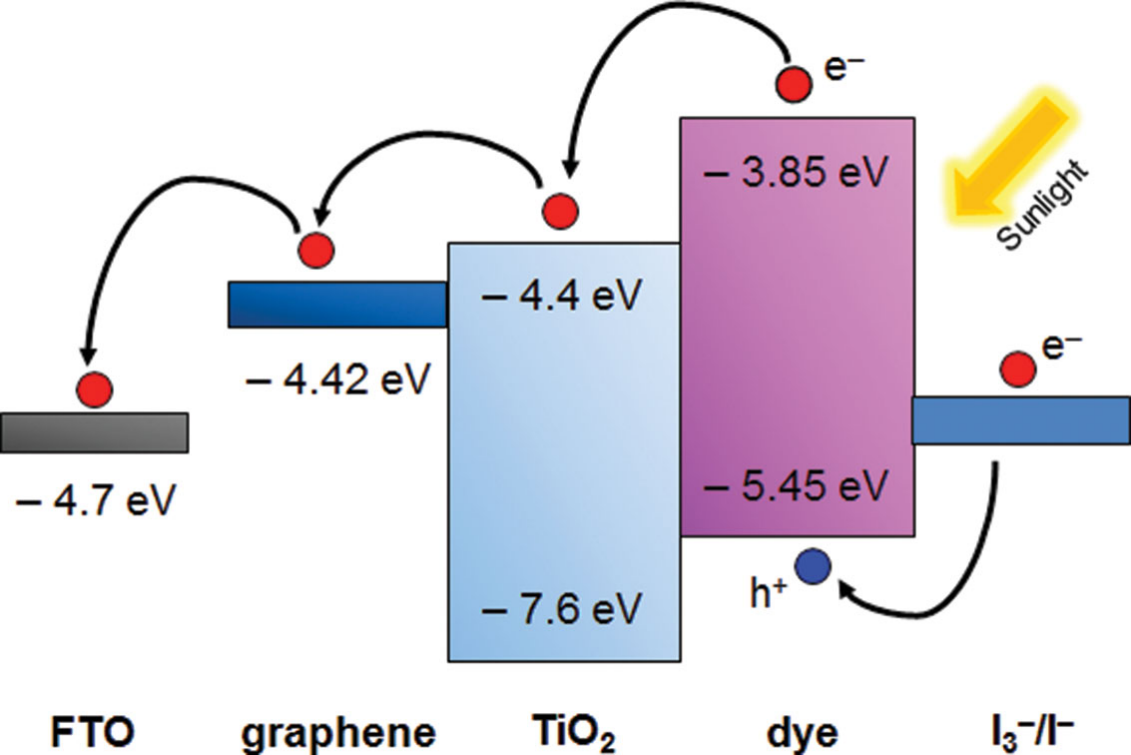
Schematic diagram of the energy level for graphene/TiO_2_ film based DSSC.

**Figure 14:**
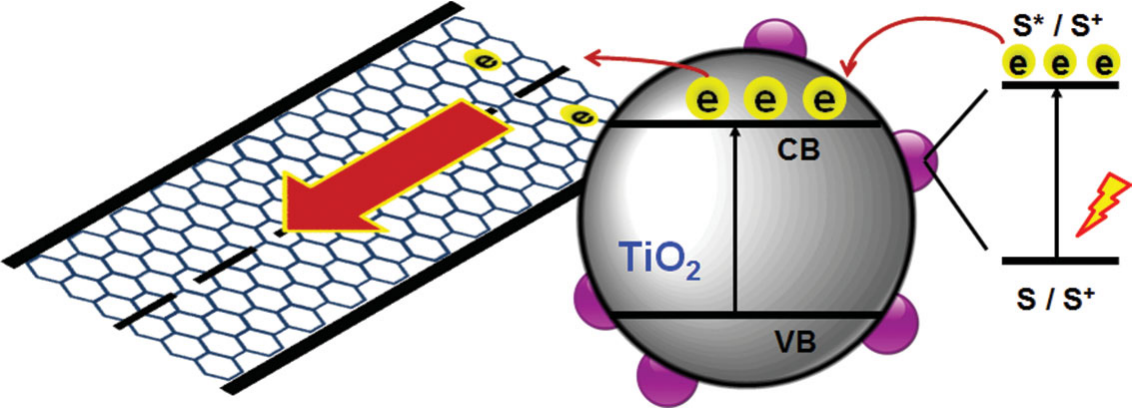
A mechanism for the enhanced electron transfer in graphene/TiO_2_.

The reasons can be listed as follows: i) For the case of CNTs, although they can improve the efficiency of DSSCs, their poorer interconnection with the spherical TiO_2_ nanoparticles (as compared to the graphene) would limit the overall performance of DSSCs due to some charge transfer barrier and possibility of recombination (see **Figure** **12**a). In contrast, graphene is a large single sheet that can significantly contact TiO_2_ nanocrystallites, thus, it would significantly suppress the charge recombination (see Figure 12b).

ii) The work function of graphene (–4.42 eV)^122^ lies between the conduction band of TiO_2_ (–4.4 eV)^13^ and FTO substrate (–4.7 eV).^13^ Owing to this suitable energy level, photo‐generated electrons transfer stepwise from the TiO_2_ to FTO without an energy barrier (see **Figure** **13**). Here, graphene can act as a bridge between TiO_2_ and FTO.

iii) The very high conductivity of graphene can accelerate the electron transporting process and reduces the rate of charge recombination (see **Figure** **14**). Because of these advantages, graphene materials have been believed to be perfect candidates for the photoelectrode of DSSCs.

To the best of our knowledge, the first study incorporating graphene materials in a TiO_2_ photoelectrode was reported by Kim et al.,^120^ who used a reduced graphene oxide/TiO_2_ nanoparticles composite as interfacial layer between the FTO and nanocrystalline TiO_2_ film. By applying this reduced graphene oxide/TiO_2_ blocking layer, they obtained an energy conversion efficiency of 5.26% which was slightly higher than that (4.89%) of the reference cell. Based on this low improvement in the DSSC performance (only 7.56%), it seems that the common TiCl_4_ treatment (TiO_2_ blocking layer) is a more effective method than using this reduced graphene oxide/TiO_2_ blocking layer. Although the enhancement in the DSSC efficiency achieved using graphene materials as blocking layer was relatively low in this work,^120^ the idea has inspired many studies to further advance this topic.

Tang et al.^122^ prepared graphene/TiO_2_ nanocomposite based photoelectrodes for highly efficient DSSCs using a molecular grafting method on titanium (IV) butoxide and graphene sheet. Because of the presence of oxygen containing functional groups on graphene, organic titanium molecules could be grafted on the functionalized graphene sheets by chemisorption. By adjusting the reduction level of graphene oxide, a good interconnection of TiO_2_ particles to the graphene sheets was achieved producing a highly conductive film. As a result, when the optimized amount of graphene was incorporated into TiO_2_ nanoparticles based film, the cell obtained five times higher efficiency than the bare TiO_2_‐based one. These authors showed that this significant improvement in the DSSC performance was due to the increased adsorption of dye molecules in the graphene/TiO_2_ film as compared to the TiO_2_‐only film. Several other studies have showed that the presence of graphene in the nanocrystalline films improves the dye loading.^124^, ^125^, ^130^ The reason for this improvement in the dye loading was explained by these authors as follows: the high surface area to volume ratio of graphene provides more anchoring sites for TiO_2_ which enable the loading of a high amount of dye molecules. In contrast, it can be seen from Table 2 that in some studies,^123^, ^126^, ^127^, ^131^ the dye loading in the TiO_2_ nanocrystallites film decreased after adding the graphene structures. Recently, Chen et al.^126^ showed that the amount of adsorbed dye in the graphene incorporated TiO_2_ film was measured to be 7.6 × 10^−9^ mol cm^−2^, which was lower than that (1.0 × 10^−8^ mol cm^−2^) of the film with only TiO_2_ nanoparticles. Furthermore, some other authors also suggested that the incorporation of graphene in TiO_2_ based film does not significantly increase the dye adsorption into the film, despite the fact that graphene with a high surface area to volume ratio was used.^123^, ^127^, ^131^ Based on this argument, it can be concluded that the high surface area to volume ratio of graphene does not completely explain the mechanism of the dye adsorption characteristic. Therefore, the kinetics of dye adsorption in graphene based films is still unclear, with some studies showing contrary results. For this reason, a deeper understanding and reasonable explanation of dye adsorption onto graphene incorporated films needs to be provided based on the careful investigations. For example, it would be reasonable to explore the amount of oxygen containing functional groups on graphene surface for the adsorption of dye molecules. Graphene is mostly synthesized by a chemical oxidation (Hummers method,^132^ followed by a chemical (by hydrazine) or a thermal reduction process. The chemically oxidized graphene involves various functional groups such as –OH, C=O, and –COOH. On the other hand, it has been reported that the functionalized graphene (graphene oxide) is capable of hydrogen bonding and π–π stacking with other organic dye molecules.^43^, ^133^, ^134^ This may mean graphene with a high number of functional groups may adsorb more dye molecules onto their surface. On the other hand, the pristine graphene has a higher electrical conductivity than the functionalized graphene. Therefore, if the functional groups on graphene play an important role in the dye loading, further investigation will be required to determine a balance between the conductivity and the dye adsorption ability of graphene oxide by optimizing the oxidation or reduction level.

It is obvious that a high concentration of graphene significantly reduces the R_ct_ of DSSCs that improves the electron transport rate, whereas this downgrades the transparency of films and thereby decreases the light harvesting efficiency of the photoelectrode. Therefore, advanced work was needed to find an optimal graphene content that benefits for both the charge recombination and light harvesting efficiency. Yang et al.^121^ synthesized graphene/TiO_2_ composites by varying the content (0–8.5 wt%) of graphene in the DSSC photoelectrodes. They found that the optimal content of reduced graphene oxide in the TiO_2_ film is ca. 0.6 wt% which is the best for cell performance. Furthermore, many studies have explored the influence of graphene content on the DSSC performance.^127^, ^128^, ^129^ It can be clearly seen from Table 2 that loading ranging from 0.6 to 0.83 wt% of reduced graphene oxide incorporated in the TiO_2_ photoelectrode films achieved the highest efficiency in the majority of studies.

**Figure 15:**
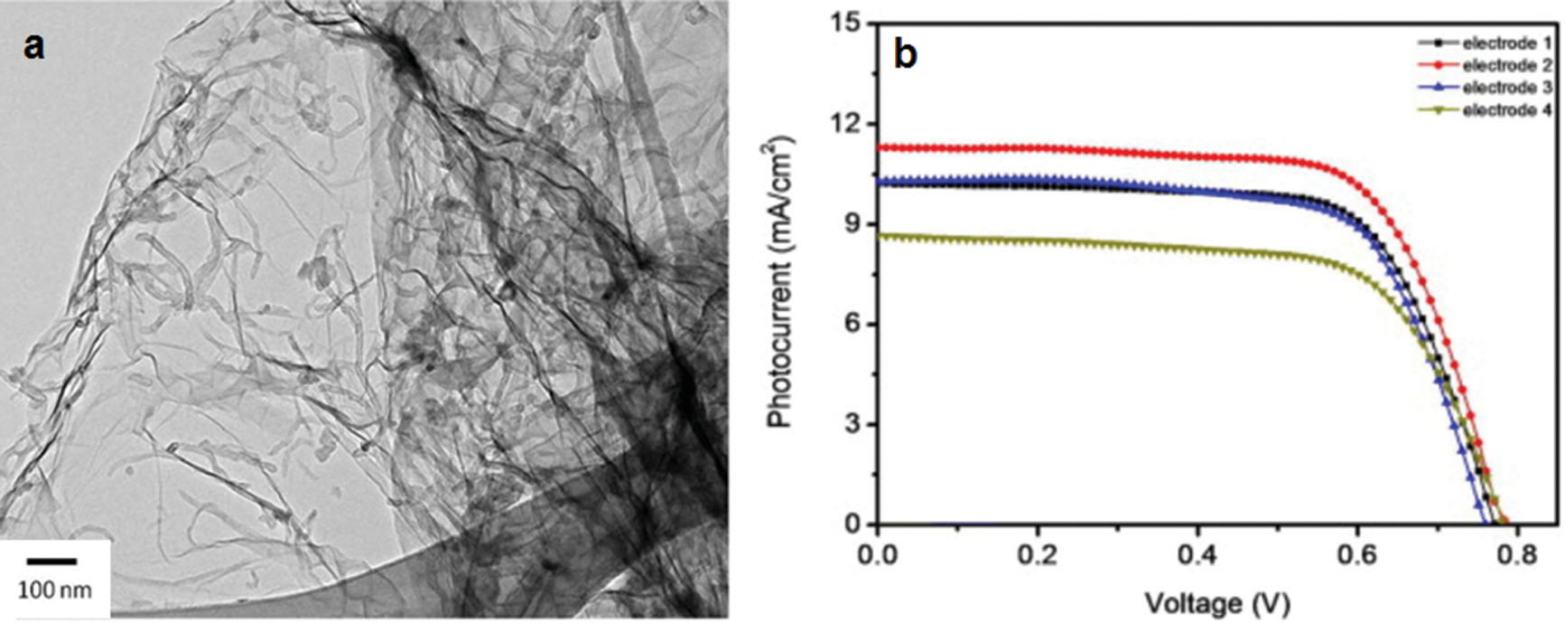
a) TEM image of MWCNTs/graphene composite and b) J–V curves of DSSCs based on acid functionalized‐MWCNTs (electrode 1), MWCNTs/graphene composite (electrode 2), graphene (electrode 3) and TiO_2_‐only (electrode 4) photoelectrodes.^136^ Reproduced with permission.^136^ Copyright 2011, Elsevier.

Due to the π–π interactions and/or hydrophobic surface of graphene layers, pristine graphene is insoluble in conventional solvents such as water and anhydrous ethanol, which is a major barrier to its successful utilization. Several noteworthy approaches have been developed to overcome this issue.^135^, ^136^ Yen and co‐workers improved the dispersion stability of graphene in an ethanol solution using MWCNTs as a spacer between graphene layers (see **Figure** **15**a) and they used the graphene/MWCNTs materials in the DSSC photoelectrodes.^136^ This 3D structured photoelectrode composed of graphene/MWCNTs/TiO_2_ nanocomposites exhibited an efficiency of 6.11%, which was significantly higher than that (4.54%) obtained by the TiO_2_‐only cell, as shown in Figure 15b. The improved performance was proven to be due to the improved dispersibility of graphene and MWCNTs in ethanol solution.

## 3 Conclusion and Future Directions

In this Review, we discussed the advanced research on the use of carbon materials in the photoelectrodes of DSSCs because the activity in this research field has been rapidly growing in the past few years. A brief overview of novel nanostructured materials based photoelectrodes is also provided. Based on the results and findings of extensive research, it can be concluded that CNTs and graphene are very promising materials for high performance photoelectrodes for DSSCs due to their fascinating properties. Although significant achievements have been made in this cutting‐edge research, several challenges must be addressed to build up high‐performance devices based on CNTs and graphene. Further optimizations of carbonaceous photoelectrodes in DSSCs are still required.

It was found that vertically aligned CNTs are promising counter electrode materials to achieve highly efficient Pt–free DSSCs due to its improved electrical conductivity and electrocatalytic activity.^77^, ^137^, ^138^ It is reasonable to expect improved performance of DSSCs by applying vertically grown CNTs structure with the TiO_2_ photoelectrode films.

s‐SWCNTs can significantly enhance the efficiency of DSSCs because of their non‐continuous band structure, whereas the m‐SWCNTs reduce the cell performance. Therefore, the bandgap of s‐SWCNTs can be tuned by controlling their defect or functionalization level, diameter and aggregation degree etc. In this regard, exploring the influence of different band structures of s‐SWCNTs on the cell performance will be an important research direction for further development of DSSCs. Similarly, chemically functionalizing graphene is an established method to open the bandgap of graphene and is critical to the improvements in the cell characteristics. The band structure of graphene oxide or reduced graphene oxide can be tuned by the level of functionalization.^139^, ^140^ The electronic band structure of the functionalized graphene should be considered in the future studies of graphene materials based DSSCs.

Since the dye adsorption kinetics on graphene structures based DSSCs are not fully understood, the underlying fundamental driving forces of dye interactions should be explored in depth. According to the literature,^43^, ^133^, ^134^ it seems reasonable that the reactive sites (functional groups) on graphene surfaces and edges would play a major roles in dye interactrions and this will need to be investigated to better understand the dye loading characteristics. If the functional groups on graphene play a critical role in the dye adsorption, further optimization of the oxidation or reduction level of graphene may be required to achieve the highest possible performance of DSSCs.

Dye lifetime is also another critical limiting factor in DSSCs.^29^ The presence of carbonaceous materials with high conductivity may help extend dye lifetimes. The use of thin films of carbonaceous material would allow the selective filtering of certain regions of the spectrum which will extend dye lifetimes.^141^, ^142^ For example, chirally sorted CNTs of particular types could be applied on the incident light side of the photoelectrode to absorb UV‐light while letting visible light pass for adsorption by the dye. The lack of UV‐light reaching the dye will enhance the active lifetime of the photoelectrode.

Furthermore, chirally sorting of the CNTs would allow the precise tuning of electronic energy levels in the electrode. This has the potential to improve performance but it will also provide avenues to investigate the exact role of CNTs in the hydrid photoelectrodes. The current understanding of the semiconducting photoelectrodes with carbonaceous structures in DSSCs is somewhat limited in terms of the exact roles of each component. Therefore, future investigations to elucidate the exact role of the various carbon materials (especially CNTs and graphene) in the photoelectrode of DSSCs will be of great value.

Some workers have also explored the effect of different types of nanotubes in the counter electrode of DSSCs.^143^, ^144^ While this review highlighted the differences between SWCNTs and MWCNTs, there seem to have been little work with double‐walled CNTs (DWCNTs). Interestingly, there is considerable work showing that DWCNTs can often provide enhanced conductivity while still providing very similar structural properties of SWCNTs.^143^, ^145^, ^146^ The use of DWCNTs in photoelectrodes is a clear research opportunity that is still to be extensively explored.

Chemical doping has been shown to be an effective method to enhance the conductivity of CNTs and graphene.^147^, ^148^, ^149^ In this regard, the use of chemically doped CNTs and graphene in the photoelectrode of DSSCs would be a valuable research direction. Additionally the use of these nanomaterials offers the exciting opportunity of nanostructuring the photoelectrode. For example, a layered structure would allow the selective, efficient harvesting of different portions of the solar spectrum as the light passed through the electrode. This affords the opportunity to make use of very high adsorbing dyes for narrow wavelength regions and this stack of high absorbers could be more efficient that the broad spectrum absorbers currently in use.

It has been shown in polymer based solar cells that beyond the electronic properties of the donor/acceptor system where functionalized CNTs are involved, the morphology also plays a key role in PV applications.^150^ For instance, the addition of functionalized CNTs to a PEDOT:PSS lowered the overall performance, but did increase the current. These changes were attributed to the nano‐morphology of the system. Recent work has demonstrated the key importance of the nanostructure of the active layer and indeed suggests light trapping in this layer could be a powerful approach to improve performance. The best structure is difficult to predict due to the competing influences of light trapping and charge conduction.^151^

Alignment within a CNT film has been demonstrated recently and offers the opportunity to both increase light transmission and film conductivity.^152^ These films offer a smooth substrate which might also be of benefit in a layered structure where direct contact between a high loading of dye molecules and the conducting element of the electrode will be possible but the current alignment approaches using highly toxic chemicals will need to be improved before this approach can be considered a serious alternative for wide scale use.

It has been demonstrated that carbon materials exhibit excellent electrocatalytic activity for the reduction of liquid electrolyte.^22^, ^29^, ^80^ However, the use of too high concentration of the carbonaceous materials in the photoelectrode brings significant charge recombination at the interface of carbons and electrolyte by reducing tri‐iodide to iodide. Therefore, the electrocatalytic activity of carbonaceous materials should be taken into account when they are used in the photoelectrode. Graphene is known not to be penetrable by gases so a film of graphene on the photoelectrode may offer an ability to control molecular diffusion while still allowing efficient charge transport. Such diffusion control might extend the lifetime of the electrolyte. This work would likely require the construction of a complex hybrid electrode perhaps using CNTs to enhance conductivity or tune electronic states while using graphene to control levels of reactivity at the critical interfaces.

We believe that the carbonaceous material will bring an important breakthrough when they are used in the photoelectrode of solid state DSSCs.
